# Supplementary material for: A high-resolution mRNA expression time course of embryonic development in zebrafish
Source: eLife. 2017 Nov 16;6:e30860. doi: 10.7554/eLife.30860 (PMC5690287; doi:10.7554/eLife.30860)
Supplement: Supplementary file 6. [file elife-30860-supp6.zip › biolayout-clusters-files/Cluster080-genes.html]

Cluster080


# Cluster080: Genes

| Ensembl ID | Gene Name | Chr | Start | End | Biotype |
| --- | --- | --- | --- | --- | --- |
| ENSDARG00000055284 | afap1 | 12 | 22702000 | 22797549 | protein\_coding |
| ENSDARG00000026531 | alcama | 10 | 28845124 | 28949177 | protein\_coding |
| ENSDARG00000056725 | hmgb3a | 7 | 25642032 | 25647088 | protein\_coding |
| ENSDARG00000003971 | isl2a | 25 | 32338903 | 32345058 | protein\_coding |
| ENSDARG00000070145 | magi1a | 8 | 53647269 | 53667665 | protein\_coding |
| ENSDARG00000028943 | mtss1la | 18 | 14801785 | 14868551 | protein\_coding |
| ENSDARG00000074143 | myo10l3 | 9 | 12690106 | 12840648 | protein\_coding |
| ENSDARG00000103379 | pax6a | 25 | 14932641 | 14953381 | protein\_coding |
| ENSDARG00000089805 | pcdh18a | 1 | 12574317 | 12584322 | protein\_coding |
| ENSDARG00000039483 | si:dkey-51a16.9 | 13 | 25298995 | 25306271 | protein\_coding |
| ENSDARG00000030125 | sox7 | 20 | 19167013 | 19178841 | protein\_coding |
| ENSDARG00000061205 | stag1a | 2 | 25363753 | 25452953 | protein\_coding |
| ENSDARG00000006120 | tbx2b | 15 | 27431518 | 27438464 | protein\_coding |
| ENSDARG00000017615 | tlx3b | 10 | 21905706 | 22087549 | protein\_coding |
| ENSDARG00000052567 | tmem35 | 14 | 40909053 | 40918138 | protein\_coding |
